# Case Report: Unusual Heterotopic Ossification of the Hindfoot

**DOI:** 10.3389/fsurg.2022.917560

**Published:** 2022-06-07

**Authors:** Falcioni Danya, Baldini Marco, Coppa Valentino, Marinelli Mario, Gigante Antonio Pompilio

**Affiliations:** ^1^ Clinical Orthopedics, Department of Clinical and Molecular Science, School of Medicine, Università Politecnica delle Marche, Ancona, Italy; ^2^ Clinic of Adult and Paediatric Orthopedic, Azienda Ospedaliero-Universitaria, Ospedali Riuniti di Ancona, Ancona, Italy

**Keywords:** heterotopic ossification, bone, foot, case report, children

## Abstract

Heterotopic ossification (HO) is a pathologic condition in which aberrant lamellar bone deposits in soft tissues, outside of the normal skeleton. Pathogenesis is still unclear, but different risk factors are known. Here we report a case of a 14 year-old girl presenting with pain in the medial calcaneal region and evidence of a rapidly growing, firm and solid neoformation. The lesion was diagnosed 6 years earlier, but it was consistently smaller and asymptomatic so that the patient did not undergo any follow up. The patient had no previous trauma or surgery, no other risk factors for HO and did not show any clinically evident HO in other districts. Xray and CT showed a heterogeneous bony lesion in the context of soft tissues, isolated from the calcaneus. After complete excision, histological analysis confirmed the diagnosis of HO. In conclusion, lone non congenital HO can occur regardless of known risk factors. Small HO lesion may also enter a proliferative phase without evidence of triggering events. More studies are required to better understand etiopathogenesis of HO in these clinical settings.

## Introduction

Heterotopic ossification (HO) is a pathologic condition in which aberrant lamellar bone deposits in soft tissues, outside of the normal skeleton. Apart from rare cases of genetic forms, the majority of HO cases are acquired and they can be either spontaneous or post-traumatic.

The early identification of patients with HO can be difficult. Clinical signs are variable and nonspecific, ranging from inflammatory signs such as pain, erythema, swelling and warmth to increased joint stiffness and limited range of motion (ROM) of the involved joint. Asymptomatic masses are not uncommon presentations, as well (1).

Here we report a case of a skeletally immature girl without any known risk factor, with a diagnosis of lone HO lesion of the calcaneal region of the right foot.

## Case Description

A 14-year-old girl presented to our hospital with pain and discomfort in the medial region of the right hindfoot and evidence of a solid and firm mass reported to be rapidly growing during the previous weeks.

She was evaluated six years earlier for a painless, slowly enlarging mass in the same region. Anamnestic evaluation at the time revealed no history of acute trauma or repetitive minor trauma, as well as a negative familiarity. Complete clinical examination confirmed the absence of other similar lesions.

**Figure 1:**
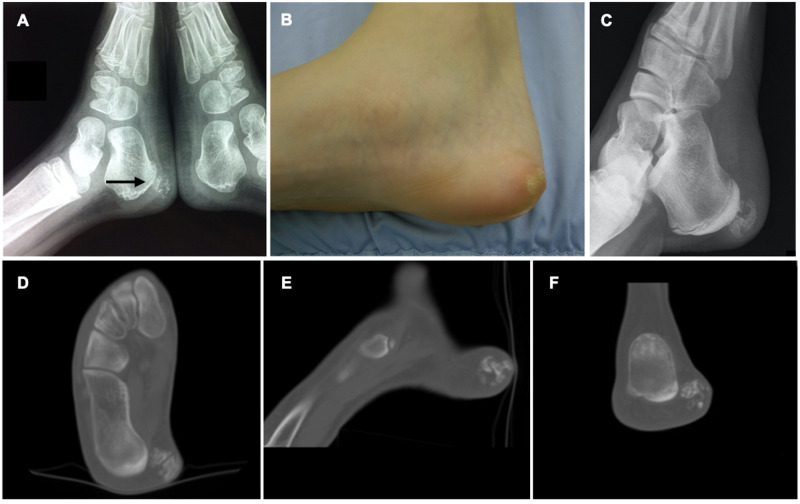
(**A**) Old X-ray showing the clinically silent lesion at the age of 6 years. (**B**) Clinical picture showing the mass covered with areas of hyperkeratosis. (**C**) New X-ray at time of presentation showed increased size of the lesion. (**D–F**) Preoperative CT section of the lesion.

Plain X-ray demonstrated a neoformation in the context of soft tissue with calcifications inside and undefined limits (Figure 1A). Ultrasound (US) had shown a poorly defined mass of mixed echogenicity measuring approximately 6 mm × 8 mm, with hyperechoic areas similar to bone signal. No cystic component or abnormal Doppler flow was found.

For approximately six years she had been completely asymptomatic and had not been doing any clinical or radiological follow up.

At the time of presentation local examination revealed moderate oedema and hyperkeratosis in the posterior-medial side of the right hindfoot with evidence of a hard, firm mass measuring about 20 mm in diameter (Figure 1B). No sign of local inflammation was evident and neurovascular examination was normal.

A new X-ray examination demonstrated that the lesion was enlarged and contained more areas of ossification (Figure 1C).

CT scan (Figures 1D–F) showed the mass to be made of mixed bone and non-ossified areas. The lesion was clearly separated from the calcaneal cortical bone and measured approximately 14 × 18 mm in its maximum diameter.

**Figure 2:**
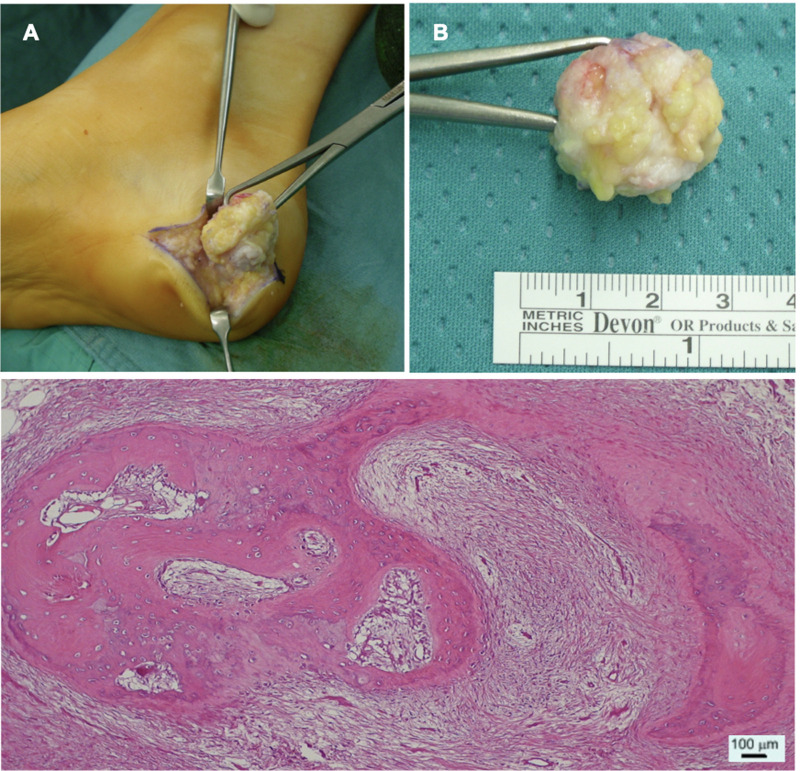
(**A**) Intraoperative image of the excision of the mass. (**B**) Picture of the sample measuring approximately 2.5 × 1.5 × 2 cm. (**C**) Section of the lesion as seen with optic microscopy and Hematoxylin & Eosin staining. The specimen shows “*mixed fibrous-adipose dermo-epidermal tissue with foci of stromal osseous metaplasia*”, as confirmed also by pathology report.

A complete excision of the mass was performed through a small lesion-centered incision (Figures 2A,B). The mass was clearly separated from calcaneal periosteum and macroscopic radical excision was achieved. Histopathological examination confirmed the lesion to be made of “multiple foci of stromal osseous metaplasia.”, thus supporting the diagnosis of HO (Figure 2C).

**Figure 3:**
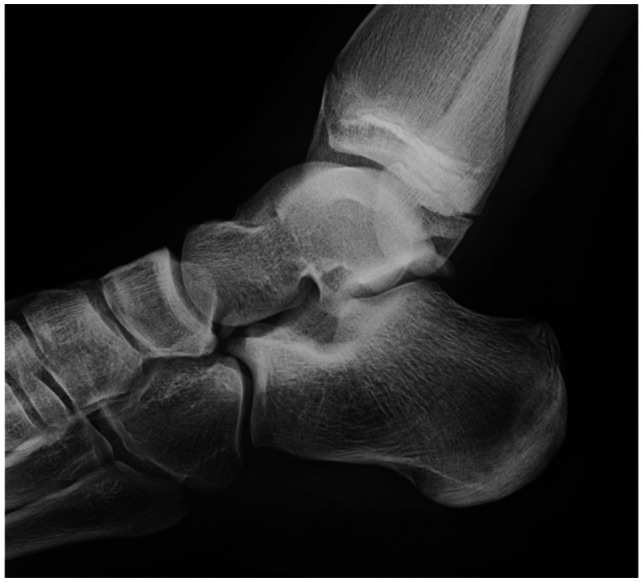
Xray 6 months after surgery confirming complete excision of the lesion without residual pathology.

Radical excision of the mass was demonstrated with Xray and confirmed during follow up (Figure 3).

No perioperative and postoperative, confirmed or suspected adverse events were recorded.

The patient was discharged without other treatments but with a strict clinical and radiological follow up, to detect early eventual local relapses or secondary lesions. During the programmed follow up the patient did not complain about local al distant symptoms in any way potentially related with relapses or secondary lesions. Thus, given the absence of secondary lesions and family history, the patient was still excluded from whole exome sequencing protocol.

**Figure 4:**
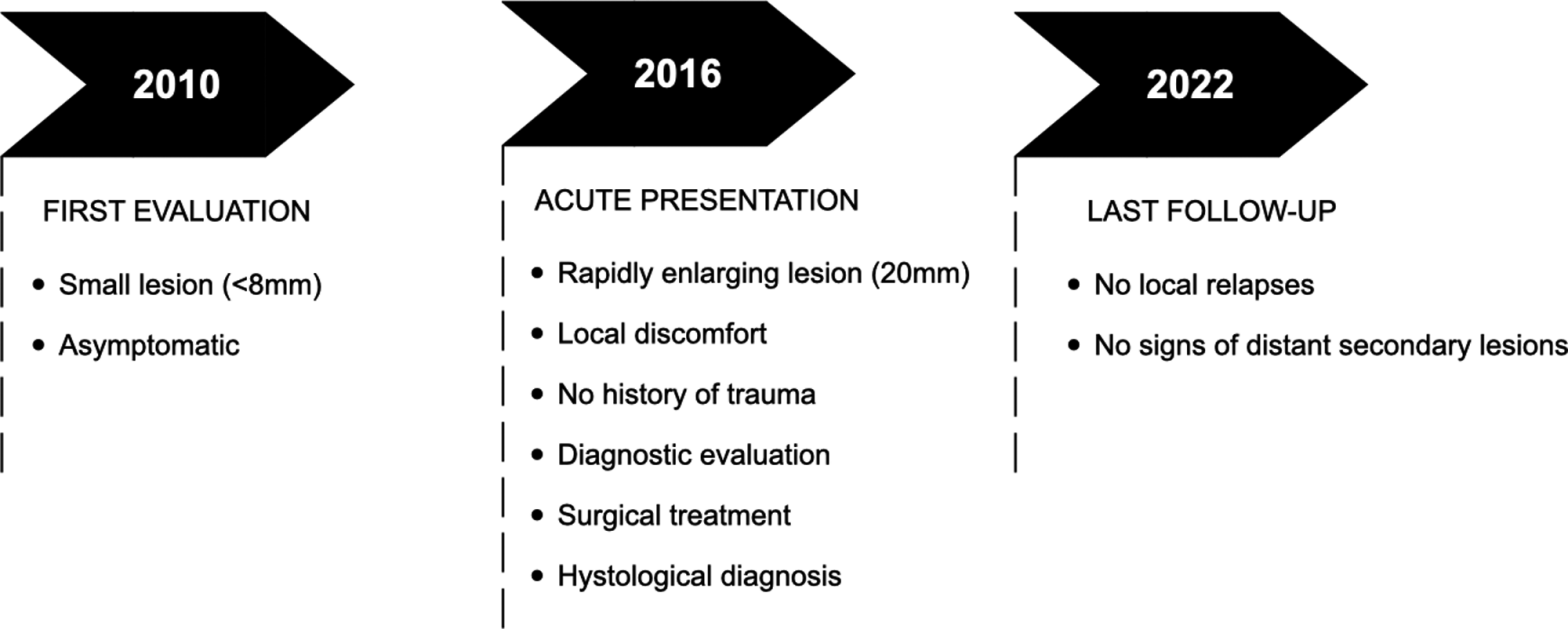
Timetable reporting relevant data from the episode of care.

Moreover, after 6 years of follow up, the patient is still asymptomatic and has experienced no signs or symptoms suggestive of local relapses or distant secondary lesions. (Figure 4 - Timetable).

## Discussion

Heterotopic ossification (HO) is a pathological condition in which qualitatively normal bone tissue aberrantly forms in extra-skeletal tissues. The ultimate pathogenetic cause is the alteration of the usual process that regulates the timing and location of bone formation. This aberrant growth takes place not within the muscle fibers, but between muscle planes. Furthermore, these lesions are isolated from the skeletal bone, and even if in contact they do not interfere with the periosteal anatomy.

The most common clinical presentation is an acquired singular neoformation in a young adult. In approximately 75% of the cases a local trauma can be identified as the triggering event and repetitive microtrauma are highly suspected in the remaining (2). The other predisposing conditions are: surgical exposures in orthopedic surgery (3), fractures or dislocations (4), spinal cord injury and other neurological and metabolic disorders (2, 5).

Rare hereditary forms include Fibrodysplasia Ossificans Progressiva (FOP) and Progressive Osseous Heteroplasia (POH), with different genetical backgrounds and pathological appearances.

African-American ethnicity was clearly recognized as an independent risk factor for development of HO after total hip arthroplasty (THA). Male sex was also suggested as an independent risk factor but in this case evidence is conflicting (6, 7).

Beyond these few known risk factors and epidemiological notes, the exact pathogenesis of heterotopic ossification is still unclear. Chalmers et al. (8) described three conditions necessary to HO formation: the recruitment of osteogenic precursor cells (9), an event that triggers the differentiation of mesenchymal cells into bone-forming cells and a suitable tissue microenvironment that supports bone formation.

Different studies have demonstrated that an insufficient inhibitory response that causes overexpression of BMP signaling (7, 8) may play a role in eliciting the differentiation of osteogenic cells. Many other cytokines have also been claimed to play a role, even though no definitive mechanisms have been identified, yet (12).

A predisposing local environment may be induced by inflammation (13), tissue hypoxia (14), alteration in peripheral nerve system (PNS) activity and neuro-inflammation (15), prolonged immobilization (16), PTH or calcitonin alterations and mismatch in Ca/P ratio (17).

Histologically, early HO lesions are characterized by a consistent number of proliferating cells that may mimic a sarcoma. Mature HO, on the other side, is clearly identified by the specific peripheral ossification pattern. These lesions are well contained in a dense fibrous pseudocapsule (18). Radiological appearance reflects histological stadium. Early non-ossified lesions are barely visible, while mature HO is clearly recognizable by the peripherical pattern of ossification, also known as “eggshell ossification”. CT is even more specific in identifying the zonal pattern (19). In contrast, MRI may sometimes be misleading due to the heterogeneous appearance, the presence of peripheral oedema and the possibility of core contrast enhancement that may pose diagnostic questions (20). Clinical signs are often consequence of local inflammation, nerve compression or stiffness. Moreover, only with a clinical examination, it is sometimes difficult to differentiate the early phase of HO from deep venous thrombosis (DVT), cellulitis, osteomyelitis or from bone-forming tumors.

Conservative treatment includes physical therapy, bisphosphonates or NSAIDs. Clinical evidence supporting the use of bisphosphonates is limited. Prophylactic radiation and indomethacin have proven to reduce the incidence of postoperative HO, especially in the setting of hip arthroplasty (1, 21).

Surgical excision is often necessary for the treatment of severe HO limiting mobility or causing pain or neurologic symptoms. The optimal timing for surgery is still controversial, but there is a general attitude towards giving importance to the clinical setting regardless the stage of the lesion. Indeed, early excision of HO is not associated with higher rates of recurrence (22).

In this report we presented a case of a lone, non-congenital and non-familiar HO lesion in the medial calcaneal region of a 14-year-old girl. After 6 years without symptoms, the patient experienced sudden onset of pain and rapid growth of the mass. The patient neither had risk factors nor evident triggering causes and was otherwise healthy and without any signs of similar lesions in other districts. She denied local trauma either of major or minor entity, local previous surgery or prolonged immobilization. Family history was completely negative for similar lesions and the patient did not report any signs or symptoms potentially related to secondary lesions. The complete excision of the mass led to complete resolution of the local symptoms. Follow up was performed as routine, and no signs of local relapses or secondary lesions were reported. After 6 years of follow up the patient is completely asymptomatic.

The major limitation of this report is the absence of a genetic evaluation. Nevertheless, this is explained by the fact that the patient presented with a single lesion, without relapses or secondary masses, without clinical suspicion of FOP, POH or Albright hereditary osteodystrophy (AHO) and with a completely negative family history. Genetics in this setting is still largely inconclusive (23, 24).

In conclusion, even though a history of trauma or microtrauma is thought to be present in almost all non-hereditary cases of HO, with this report we underline the importance of considering this diagnosis even when the anamnestic feature of trauma is absent. In fact, this lesion could be identified as idiopathic. In addition, previous silent HO lesion may enter in a sort of proliferative phase even without evidence of trauma or other anamnestic risk factors.

## Data Availability

The raw data supporting the conclusions of this article will be made available by the authors, without undue reservation.

## References

[1] Meyers C Lisiecki J Miller S Levin A Fayad L Ding C Heterotopic ossification: a comprehensive review. JBMR Plus. (2019) 3(4):e10172. 10.1002/jbm4.10172 31044187 PMC6478587

[2] Teasell RW Mehta S Aubut JL Ashe MC Sequeira K MacAluso S A systematic review of the therapeutic interventions for heterotopic ossification after spinal cord injury. Spinal Cord. (2010) 48(7):512–21. 10.1038/sc.2009.175 20048753 PMC3218076

[3] Riegler HF Harris CM. Heterotopic bone formation after total hip arthroplasty. - Cerca con Google. https://www.google.com/search?q=Riegler+HF%2C+Harris+CM.+Heterotopic+bone+formation+after+total+hip+arthroplasty.&rlz=1C5CHFA_enIT859IT859&oq=Riegler+HF%2C+Harris+CM.+Heterotopic+bone+formation+after+total+hip+arthroplasty.&aqs=chrome..69i57.553j0j4&sourceid=chrome&ie=UTF-8 (Accessed December 17, 2020). 1277668

[4] Hong CC Nashi N Hey HW Chee YH Murphy D. Clinically relevant heterotopic ossification after elbow fracture surgery: a risk factors study. Orthop Traumatol Surg Res. (2015) 101(2):209–13. 10.1016/j.otsr.2014.10.021 25701160

[5] Licini C Farinelli L Cerqueni G Hosein A Marchi S Gigante A Heterotopic ossification in a patient with diffuse idiopathic skeletal hyperostosis: input from histological findings. Eur J Histochem. (2020) 64(4):317–22. 10.4081/EJH.2020.3176 PMC7731577 33272008

[6] Davis G Patel RP Tan TL Alijanipour P Naik TU Parvizi J. Ethnic differences in heterotopic ossification following total hip arthroplasty. Bone Jt J. (2016) 98-B(6):761–6. 10.1302/0301-620X.98B6.36050 27235517

[7] Slone HS Walton ZJ Daly CA Chapin RW Barfield WR Leddy LR The impact of race on the development of severe heterotopic ossification following acetabular fracture surgery. Injury. (2015) 46(6):1069–73. 10.1016/j.injury.2015.01.039 25744171

[8] Observations on the induction of bone in soft tissues - PubMed. https://pubmed.ncbi.nlm.nih.gov/1090627/ (Accessed December 31, 2020). 1090627

[9] Pignolo RJ Shore EM. Circulating osteogenic precursor cells. Crit Rev Eukaryot Gene Expr. (2010) 20(2):171–80. 10.1615/CritRevEukarGeneExpr.v20.i2.70 21133846 PMC3753686

[10] Bosch P Musgrave D Ghivizzani S Latterman C Day CS Huard J. The efficiency of muscle-derived cell-mediated bone formation. Cell Transplant. (2000) 9(4):463–70. 10.1177/096368970000900403 11038063

[11] Yano M Kawao N Okumoto K Tamura Y Okada K Kaji H. Fibrodysplasia ossificans progressiva-related activated activin-like kinase signaling enhances osteoclast formation during heterotopic ossification in muscle tissues. J Biol Chem. (2014) 289(24):16966–77. 10.1074/jbc.M113.526038 24798338 PMC4059139

[12] Shore EM Kaplan FS. Role of altered signal transduction in heterotopic ossification and fibrodysplasia ossificans progressiva. Curr Osteoporos Rep. (2011) 9(2):83–8. 10.1007/s11914-011-0046-3 21340697 PMC3433752

[13] Hsieh HHS Chung MT Allen RM Ranganathan K Habbouche J Cholok D Evaluation of salivary cytokines for diagnosis of both trauma-induced and genetic heterotopic ossification. Front Endocrinol. (2017) 8. 10.3389/fendo.2017.00074 PMC5401868 28484423

[14] Agarwal S Loder S Brownley C Cholok D Mangiavini L Li J Inhibition of Hif1α prevents both trauma-induced and genetic heterotopic ossification. Proc Natl Acad Sci U S A. (2016) 113(3):E338–47. 10.1073/pnas.1515397113 26721400 PMC4725488

[15] Kaplan FS Pignolo RJ Shore EM. Granting immunity to FOP and catching heterotopic ossification in the Act. Semin Cell Dev Biol. (2016) 49:30–6. 10.1016/j.semcdb.2015.12.013 26706149 PMC4898187

[16] Hudson SJ Brett SJ. Heterotopic ossification - A long-term consequence of prolonged immobility. Crit Care. (2006) 10(6):174. 10.1186/cc5091 17129365 PMC1794459

[17] Fournier DE Kiser PK Beach RJ Dixon SJ Séguin CA. Dystrophic calcification and heterotopic ossification in fibrocartilaginous tissues of the spine in diffuse idiopathic skeletal hyperostosis (DISH). Bone Res. (2020) 8(1). 10.1038/s41413-020-0091-6 32257530 PMC7118090

[18] Hoda SA. Enzinger and Weiss’s soft tissue tumors, 6th edition. Adv Anat Pathol. (2014) 21(3):216. 10.1097/pap.0000000000000020

[19] Kransdorf MJ Meis JM. From the archives of the AFIP. Extraskeletal osseous and cartilaginous tumors of the extremities. Radiographics. (1993) 13(4):853–84. 10.1148/radiographics.13.4.8356273 8356273

[20] Kransdorf MJ Meis JM Jelinek JS. Myositis ossificans: MR appearance with radiologic-pathologic correlation. Am J Roentgenol. (1991) 157(6):1243–8. 10.2214/ajr.157.6.1950874 1950874

[21] Popovic M Agarwal A Zhang L Yip C Kreder HJ Nousiainen MT Radiotherapy for the prophylaxis of heterotopic ossification: a systematic review and meta-analysis of published data. Radiother Oncol. (2014) 113(1):10–7. 10.1016/j.radonc.2014.08.025 25220370

[22] Chalidis B Stengel D Giannoudis PV. Early excision and late excision of heterotopic ossification after traumatic brain injury are equivalent: a systematic review of the literature. J Neurotrauma. (2007) 24(11):1675–86. 10.1089/neu.2007.0342 18001198

[23] Yang Z Liu D Guan R Li X Wang Y Sheng B. Potential genes and pathways associated with heterotopic ossification derived from analyses of gene expression profiles. J Orthop Surg Res. (2021) 16(1):1–10. 10.1186/S13018-021-02658-1/FIGURES/7 34389038 PMC8364104

[24] Cappato S Gamberale R Bocciardi R Brunelli S. Genetic and acquired heterotopic ossification: a translational tale of mice and men. Biomedicines. (2020) 8(12):611. 10.3390/BIOMEDICINES8120611 PMC7765130 33327623

